# The Open Access Movement Grows Up: Taking Stock of a Revolution

**DOI:** 10.1371/journal.pbio.1001686

**Published:** 2013-10-22

**Authors:** Heather Joseph

**Affiliations:** SPARC (The Scholarly Publishing and Academic Resources Coalition), Washington, DC, United States of America

## Abstract

Heather Joseph, Executive Director of SPARC, contributes to our Tenth Anniversary *PLOS Biology* Collection by discussing how the Open Access movement has grown up.

## Introduction

It's been just over a decade since the concept of Open Access (OA) first captured the attention of the scientific and scholarly research community, bringing with it the promise and potential of a shining new digital landscape, in which knowledge is freely shared and freely used, and the pace of scientific discovery is accelerated for the benefit of all.

Early meetings, convened by diverse groups of thought leaders around the world, resulted in a handful of key Declarations that provided a strong intellectual and philosophical foundation for the movement, and also reflected the convergence of opportunities that allowed scientists to consider a completely new way of sharing information. As the participants in one such key meeting, the Budapest Open Access Initiative (BOAI), noted in 2002:

*An old tradition and a new technology have converged to make possible an unprecedented public good*… [1]

The tradition referred to by the BOAI is the longstanding practice of scholars to publish papers in journals without expectation of payment; the new technology is, of course, the Internet. The idea that these two elements could be seamlessly combined to ensure that anyone, anytime, anywhere in the world could have the ability to immediately and freely access the results of scholarly research online ignited a firestorm of excitement in many quarters across the academy.

Early supporters of the concept quickly recognized its potential to transform the research process. Not only would it allow them to access tens of thousands of articles that were previously unavailable for them to read, but it would also allow them to use these digital articles in previously unimaginable ways. Rather than being constrained to reading one article at a time, hopping from siloed website to siloed website, scholars could now envision a world where articles could be used in bulk and treated as digital data. They would now have the opportunity to download a significant corpus of the literature, run computational or data mining technologies, and facilitate entirely new ways of using scholarly articles.

Scholars also recognized the extraordinary potential that OA held for authors to open up their work to vast new audiences across disciplinary and geographic boundaries, offering the chance to gain new readers and allowing significant and measurable increases in the visibility and impact of their work. This increased access also had significant implications outside of research labs, democratizing the ability of educational institutions to access high-quality information and providing a new channel for businesses, entrepreneurs, and interested members of the public—in many cases, for the first time.

In the view of many, OA provided a compelling vision of the future of research communication, and one that was ripe with promise. This spurred some early community declarations of support, including a notable petition sponsored by the Public Library of Science (PLOS) that was signed by more than 30,000 individuals who collectively declared their intent to act in support of OA practices [2].

With such a compelling vision and with such clear, tangible benefits serving as drivers, a wholesale move to an OA system should have been easy, no? Not quite—as with any significant movement for change where there are significant societal, ethical, and financial outcomes at stake, there is no such thing as an overnight success.

Implementing such wholesale changes in the context of traditional scholarly journal publishing proved to be an extraordinarily challenging venture. A complex set of interwoven factors—from copyright transfer routines that indiscriminately transferred limitations from a paper-based world to the digital environment, to evaluation practices that have intimately tied funding, tenure, and promotion decisions to publication in established flagship “high-end” journals, and not to mention the fact that scholarly publishing is a multibillion dollar revenue-generating industry [3] with absolutely no interest in ceding their claim on this layer of content—came at times to resemble a Gordian knot that at times seemed impossible to unravel.

Yet, despite these substantial obstacles, over the past decade, we have seen the acceptance and adoption of OA grow, steadily and inexorably, year in and year out. How has this been possible? It's tempting, when writing a retrospective, to simply point to a person or an event (or set of them) and say: these were the catalysts; this was the precipitating event that made it all happen. And while OA, of course, does have its notable leaders and benchmark events, the collective movement has always been greater than the sum of its parts.

This was driven home to me at a recent meeting in Mexico City. Public Knowledge Project founder John Willinsky recounted the experiences he'd had a decade ago, traveling throughout Latin America and working to promote the idea of the academic community taking back control of their research output by using OA. “Sometimes there'd be 15 people in the room, and sometimes a few hundred,” he noted. “But I was so sure that at the end of each discussion, *all* of them would rush up to me and say, ‘Sign me up! I'm in.’” He recalled his disappointment when, time and time again, that simply didn't happen, and he found himself, as he continued his work over the subsequent years, regularly wondering if anyone was listening at all.

Returning to Mexico this year, almost exactly 10 years later, he had his answer: a thriving culture of OA has emerged, with academic and research institutions supporting the creation and publication of several thousand OA journals via platforms like SCIELO and Redalyc, and a thriving network of open digital repositories.

As I listened to him speak, it occurred to me that his experience mirrored not only my own but also those of countless others during the last decade. Whether on a campus or in a research lab, in the office of a co-author or a faculty advisor, in a conversation with a policy maker or a research funder, or even just in our own organization with our own colleagues, this simple process of making the case for OA, of planting the seed through education and advocacy, has played out again and again, thousands of times around the world. For me, the story of the last 10 years has truly been the story of these myriad individual actions building one upon another, resulting in a full-fledged global movement making OA the norm in how we share research and scholarship.

Over the past decade, the OA movement has both expanded and matured. Although there's certainly no official “Open Access advocate's checklist” that we've been working from to help us measure and mark our progress, a few areas stand out as places where can we truly measure how far we've progressed.

## We've Imagined a Better Future

The strong, consistent definition of OA, established in the Declarations noted earlier in key meetings in Budapest, Berlin, Bethesda, and Salvador de Bahia, provides the community with a vision of an ideal “end game” to collectively aspire to. The need for the free, immediate availability on the open Internet of articles reporting research results, coupled with the rights to fully use these articles in the digital environment, has become the widely established, fully accepted goal of the global OA movement.

Because of this strong foundation, it is now a widely held belief that until its results are communicated as broadly as possible, a piece of research is only half completed. This clear vision has helped to set the stage for the kind of consistent—and measurable—progress we've seen over the past decade towards making OA a reality.

## We've Built a Robust Infrastructure

To move OA from the theoretical to the practical, we need a solid infrastructure in place to support it. We can look to four key pieces of infrastructure that provide excellent indicators of just how far the OA movement has come in successfully implementing a truly open ecosystem for communicating research and scholarship:

The first is the establishment of a robust set of OA journals. The Directory of Open Access Journals (DOAJ) currently lists nearly 10,000 fully OA journals [4], from around the globe and in a vast variety of disciplines. As groundbreaking publishers (notably PLOS) have led the way in demonstrating that OA journals can be both high quality and financially sustainable. Over the past decade, submissions to OA journals, while starting off relatively slowly, have begun to accelerate at a rapid pace [5], and the discussion today centers on when—not if—OA will become the dominant form of journal publishing [6].

A second crucial piece of the infrastructure is the establishment of OA repositories. Higher education institutions, research facilities, funders, and governments have established more than 2,000 [7] repositories around the globe. This provides an important mechanism to ensure that research articles, along with research data and other important research outputs, can be housed, networked, curated, and sustainably archived, and also ensures that they can be accessed not only by this generation of researchers, but by future generations as well.

A third requirement is the consistent employment of open licenses. For OA to achieve both of its aims—accessibility and utility—we need the flexibility to operate within the current copyright environment to allow digital articles to be fully accessed and reused. The adoption of Creative Commons licenses (particularly the CC-BY license) has been steadily, albeit slowly, climbing [8]. While much more work remains to be done here, the trend line for adoption of these licenses is demonstrably moving in the right direction.

A fourth and final piece of infrastructure is the adoption and implementation of policies supporting OA. From being nonexistent just ten short years ago, dozens [9] of policies have now been established on the campus level, largely driven by the grassroots efforts of faculty, students, and librarians. These campus-based policies have played a key role in demonstrating that OA is consistent with—and even essential to—achieving the core mission of higher education institutions,

On the national level, funder-based policies designed to fully leverage both the public and private sectors' investment in research by providing OA to research outputs have begun to rapidly proliferate around the world, sending perhaps the strongest signal yet that the underlying rules of the scholarly communication game are undergoing a sea change.

## We're Building the Global Community

By any measure, the infrastructure needed to support OA has blossomed in the past decade. But of course, infrastructure is truly valuable only when it is fully put to use. It's difficult to point to mechanisms that accurately measure the growth of the global community who actively consider themselves part of the OA movement, but some useful indicators are out there. We can look at the geographic diversity of authors submitting and publishing papers in OA journals as one indicator, or the proliferation of national OA policies in countries ranging from Argentina to Australia as another.

But as John Willinsky's story alluded to earlier, though these indicators of community might be hard to measure, it's not impossible to do so. Just five years ago, SPARC and Students for Free Culture thought the people who were committed to promoting the idea of OA might find it useful to have a day set aside where OA education and advocacy could actively be encouraged. They held a “National Day for Open Access” in October of 2007, which garnered participation by just 12 campuses in the US. However, the event piqued the interest of potential participants around the world, and just two short years later, demand was so strong that the event was expanded into the current “Global Open Access Week” event, which last year included participation by tens of thousands of individuals, and events in 130 countries around the globe. OA community growth has been both organic and exponential.

## We've Begun to Accelerate the Culture Change Needed to Make OA the Norm

The growth of the community has been encouraging, but there is still a long way to go toward making OA the norm. In recent years, social media has helped to accelerate education and advocacy efforts, providing new platforms for the community to leverage efforts that used to be, by nature, one-off conversations. The power of channels like Twitter, Reddit, and Facebook to amplify conversation, encourage action, and establish a sense of community has been invaluable in raising awareness.

However, we've learned that simply making people aware of the potential of OA is often not enough to spur changes in behavior. In order to encourage the adoption of OA practices—publishing in OA journals, depositing in OA repositories, choosing an open license, etc.—requires concrete incentives.

Perhaps the single biggest development that has the potential to accelerate behavior change and grow the community is the development of Article-Level Metrics. The emergence of a novel set of measures that can be applied in a transparent manner to individual articles to accurately assess their importance—and impact—has strong appeal to authors and research evaluators alike.

As noted earlier, one of the biggest barriers to encouraging a wholesale move toward publishing in an OA journal has been the concern that this behavior won't be rewarded in the evaluation processes for funding, tenure, and promotion. These important assessments have come to rely heavily on citation indicators as the preferred metric. However, once evaluators have a broader palette of article- or author-level indicators to work from, they'll be able to paint a much more robust picture of impact. As these new metrics are put to use by and become more widely accepted as measures in evaluation processes, chances are good that authors will become much more willing to publish in OA outlets.

Overall, the story of the OA movement over the past ten years has been one of demonstrable progress. To be sure, the road has not been a smooth one. There have been stumbles, wrong turns, false starts, and bruising battles, particularly in the policy arena. But if we weigh the indicators of progress made by the OA movement against the intensity and complexity of the obstacles it has faced in the first decade, there's reason for great optimism as we head into the next ten years.
